# Spatial distribution and associated factors of poor tetanus toxoid immunization among pregnant women in Ethiopia: spatial and multilevel analysis

**DOI:** 10.3389/fgwh.2023.1138579

**Published:** 2023-09-04

**Authors:** Desale Bihonegn Asmamaw, Wubshet Debebe Negash, Fantu Mamo Aragaw, Habitu Birhan Eshetu, Melaku Hunie Asratie, Tadele Biresaw Belachew

**Affiliations:** ^1^ Department of Reproductive Health, Institute of Public Health, College of Medicine and Health Sciences, University of Gondar, Gondar, Ethiopia; ^2^ Department of Health Systems and Policy, Institute of Public Health, College of Medicine and Health Sciences, University of Gondar, Gondar, Ethiopia; ^3^ Department of Epidemiology and Biostatistics, Institute of Public Health, College of Medicine and Health Sciences, University of Gondar, Gondar, Ethiopia; ^4^ Department of Health Education and Behavioral Sciences, Institute of Public Health, College of Medicine and Health Sciences, University of Gondar, Gondar, Ethiopia; ^5^ Department of Women’s and Family Health, School of Midwifery, College of Medicine and Health Sciences, University of Gondar, Gondar, Ethiopia

**Keywords:** tetanus toxoid, immunization, spatial, Ethiopia, EDHS

## Abstract

**Background:**

Neonatal mortality from tetanus can be reduced by 94% when pregnant women receive at least two doses of tetanus toxoid. In Ethiopia, immunization programs are suboptimal despite their importance. Therefore, the aim of this study was to examine the geographic distribution and associated factors of poor tetanus toxoid (TT) immunization among pregnant women in Ethiopia.

**Methods:**

Secondary data analysis was used using the 2016 Ethiopian Demographic and Health Survey (EDHS). ArcGIS version 10.8 statistical software was used to explore the spatial distribution of poor TT immunization and SaTScan version 9.6 software was used to identify significant hotspot areas of poor TT immunization. For associated factors, a multilevel binary logistic regression model was fitted using STATA version 14 software. In the multivariable multilevel analysis, adjusted OR (AOR) with 95% CI was reported to reveal significantly associated factors of poor TT immunization.

**Result:**

In Ethiopia, the spatial distribution of poor tetanus toxoid immunization was clustered with Global Moran's *I* = 0.59 at *p*-value of <0.0001. The highest poor TT immunization clusters were observed in the East and South Tigray, the central part of Amhara, West Afar, East Somali, and West Gambella. Pregnant women with no Antenatal care (ANC) visits [Adjusted Odds Ratio (aOR) = 10.46, 95% CI: (8.82, 12.41))], pregnant women with 1–3 ANC visits [aOR = 1.51, 95% CI: (1.31, 1.73)], media exposure [aOR = 1.45, 95% CI: (1.26, 1.67)], poor wealth index [aOR = 1.22; 95% CI: (1.03, 1.45)], middle wealth index [aOR = 1.23; 95% CI: (1.03, 1.47)], family planning use [aOR = 1.28; 95% CI: (1.11, 1.57)] and community level education [aOR = 1.43, 95% CI: (1.14, 1.80)] were significantly associated with poor tetanus toxoid immunization.

**Conclusion:**

Poor tetanus toxoid immunization among pregnant women varies in Ethiopia. It was highest in East and South Tigray, the central part of Amhara, West Afar, East Somali, and West Gambella. Therefore, public health programs should design targeted interventions in identified hot spots to improve tetanus toxoid immunization. Health programmers should be promoting optimal ANC visits, women's education, and family planning use.

## Introduction

Maternal and Neonatal Tetanus (MNT) has been among the most common life-threatening consequences of unclean deliveries and umbilical cord care practices, and are indicators of inequity in access to immunization and other maternal, newborns, and child health services (1, 2). Every year, 3.3 million newborns die from neonatal tetanus, which accounts for a large proportion, particularly in developing countries like Ethiopia where home deliveries are common (3). Approximately 15,000–30,000 maternal mortality is caused by tetanus related to delivery every year (4). The World Health Organization (WHO) report states that the majority (90%) of maternal and neonatal tetanus cases occurred in South East Asian (SEA) and Sub-Saharan African (SSA) countries, and most of them ended with death (5).

The primary strategies for preventing MNT at birth were immunization of mothers with a protective dose of tetanus toxoid (TT) and clean delivery (6, 7). Tetanus toxoid vaccination (TT2+) can reduce neonatal mortality related to tetanus by nearly 94% in women of childbearing age (8). According to the WHO, women of childbearing age should take five successive doses of tetanus toxoid (TT) for lifelong protection (1).

During antenatal care appointments, pregnant women can receive at least two doses of the TT vaccine in different countries. But among expectant mothers, the coverage of TT2+ vaccinations ranged between 27% to 71% (9, 10). It indicates that the majority of the countries were not meeting the WHO's global immunization target of at least 90% national TT vaccine coverage and at least 80% coverage in every district (11). Despite the fact that Ethiopia is expected to reach 86% coverage of national tetanus protection at birth by 2015, only 48% of mothers in 2011% and 49% of mothers in 2016 were protected (12).

Age of the mothers, educational status of the women, marital status, occupational status, joint health decision with husband, distance from health facilities, wealth index, fear of side effects, fear of sterility, ANC follow-up, parity, lack of information about TT vaccination, knowledge, attitude, and low awareness of mothers were significant factors leads to poor TT immunization countries (2, 6, 10, 13–15). TT immunization status also differs from urban to rural mothers as well as from region to region in different countries (14, 16).

Various studies have been conducted in Ethiopia to identify associated factors for TT immunization. The spatial distribution of TT immunization was unclear in regions of Ethiopia. Understanding the level and geographical variation of TT immunization in Ethiopia can help health planners, programmers, partners in the health sector, and policymakers formulate appropriate strategies and interventions and provide quality reproductive health services to increase TT immunization. Hence, this study aimed to assess the spatial distributions of TT immunization and associated factors in Ethiopia using Ethiopian Demographic and Health Survey (EDHS) datasets.

## Methods

In Ethiopia, there are nine regions [Afar, Tigray, Amhara, Oromia, Somali, Southern Nations, Nationalities, and People's Region (SNNPR), Benishangul Gumuz, Gambella, and Harari] and two administrative cities (Addis Ababa and Dire Dawa) (17). Based on Worldometer's analysis of the latest United Nations data, Ethiopia has 121,989,792 citizens as of Thursday, December 22, 2022 (18).

A secondary data analysis was undertaken using the 2016 Ethiopian Demographic and Health Survey (EDHS), which was a nationwide representative sample that was carried out from January 18 to June 27, 2016. Once permission had been obtained by securing an online request after explaining the purpose of the study, the EDHS 2016 was accessed from the DHS official database, www.measuredhs.com. Using the 2007 Population and Housing Census (PHC) as a sampling frame, a cross-sectional study design was used with stratified cluster sampling. A stratification was carried out by separating Ethiopia's nine regional states and its two city administrations, then by dividing it into urban and rural areas (17).

A total of 645 Enumeration Areas (EAs) (202 in urban areas and 443 in rural areas) proportional to EA size were selected proportionally to the EA size in the first stage. In the second stage, 28 homes from each cluster (EA) were chosen systematically with an equal chance (12). For this study, the study population was women (aged 15–49 years) who had a pregnancy five years before the survey. We used individual datasets. A total weighted sample of 7,397 pregnant women were included in the present study**.** Additionally, latitude and longitude coordinates were taken from selected EAs (clusters). Full details of the EDHS sampling system were presented in the report (17, 19).

### Study variables

This study's outcome variable was poor TT immunization, which is defined as poor TT immunization if pregnant women did not receive enough immunization (did not receive two and above tetanus toxoid vaccine) and otherwise no. Depending on different literature reviews, individual and community-level variables were included in the analysis. Age of the women (15–24, 25–34, and 35–49), women's education (no formal education, primary education, and secondary education and above), occupation (employed, not employed), modern family planning use (yes, no), parity (primipara, multipara, grand multipara) and number of antenatal care (ANC) visits (0, 1–3, and ≥4), person who decides on respondents health care (self, jointly, and husband alone) were considered as individual-level variables (20–27). Media exposure; those who read newspapers, listened to the radio, or watched television at least once a week were coded yes and no otherwise (14). The EDHS assessed the wealth index based on 28 data items on household-selected assets, water, and toilet access used to generate the wealth status of the household, and the household wealth index status was done using principal component analysis (PCA). The overall household data items are grouped into nine items: ownership of the house, types of living house floors, walls, roof materials, household properties, types of toilets, types of fuel mainly used for cooking, the main source of water, household animal type and number, farming land ownership, and agricultural product item and amount. To categorize individuals into wealth quintiles (poor, middle, and rich), we used household asset data via principal component analysis (PCA) (28). Wanted last child (wanted no more, wanted later, and wanted then). Wanted last child indicates whether the woman wanted the last child (previous child), wanted to give birth after two years, or did not want to give birth throughout her life. Wanted then means women who wanted to deliver within two years for the last child, wanted later means women who wanted to postpone for at least two years, and wanted no more means women who did not want any more children throughout their life.

Of the community level factors, distance to the health facilities (big problem, not big problem), residence (rural, urban), and in accordance with a previous study conducted in Ethiopia and based on its geopolitical features, region was divided into three regions: small peripheral regions (Afar, Somalia, Benishangul, and Gambela), large central regions (Tigray, Amhara, Oromia, and Southern Nations Nationalities and Peoples Region), and metropolitan (Harari, Dire Dawa, and Addis Ababa) were directly accessed from EDHS data set (29–31). However, the aggregate community-level independent variables (community-level poverty, community-level media exposure, and community-level education) were constructed by aggregating individual-level characteristics at the community (cluster) level. They were classified as high or low based on the distribution of the proportion values calculated for each community after examining the distribution using the histogram. Since the aggregate variable was not normally distributed, the categorization was based on the median value (25, 32, 33).

### Data management and analysis

For data analysis, we used STATA 14, ArcGIS 10.8, and SaTScan 9.6 software. For the analysis, sample weights were applied to adjust for the non-proportional sampling of strata and regions during the survey process and to restore representativeness. Text, figures, and tables were used to present descriptive statistics and summary statistics (17, 33).

### Spatial analysis

#### Spatial autocorrelation analysis

The presence of spatial autocorrelation was identified using Moran's index (Moran's I). A Moran's I value close to −1 indicates that disease/events are dispersed, whereas a Moran's I value close to +1 indicates that they are clustered, and a Moran's I value of zero indicates that they are distributed randomly. There was a significant Moran's I (*p* < 0.05), indicating the presence of spatial autocorrelation and rejecting the null hypothesis (poor TT immunization is randomly distributed). Hotspot analysis was conducted using the Getis-Ord Gi* statistic (17).

#### Spatial interpolation

The spatial interpolation was done to predict poor immunization in unsampled areas of the country based on sampled measurements. Ordinary Kriging (OK) and Empirical Bayesian Kriging (EBK) were done since they statistically optimized the weight to predict the prevalence of poor immunization in the unobserved areas based on the observed measurement. The ordinary Kriging spatial interpolation method was selected for this study for predictions of poor immunization coverage since it had a smaller residual and Root Mean Square Error (RMSE) than EBK.

#### Spatial scan statistical analysis

Spatial scan statistics applied using Kulldorff's SaTScan software identified statistically significant primary (most likely) and secondary clusters of poor TT immunization. In SaTScan™ works, a window moves across the study areas and the window size needs to be fixed. As the outcome variable was Bernoulli distribution, Kulldorff's method was applied to use a Bernoulli model for a purely spatial analysis. In order to fit the Bernoulli model, respondents with poor TT immunization were considered case, and those with high TT immunization were considered control. Using the default maximum spatial cluster size of 50% of the population as an upper limit, both small and large clusters were detected, and clusters with more than the maximum level were ignored. Poor TT immunization was considered in areas with a high Log Likelihood Ratio and significant *p*-value compared to areas outside the window.

### Multilevel analysis

Descriptive statistics were described using frequency and percentage. A variance inflation factor (VIF) was used to test for multicollinearity, and a VIF of less than five was obtained for each independent variable, with a mean VIF of 1.47, indicating there was no significant multicollinearity between independent variables. In the EDHS data, there was a hierarchical structure, which violates the independent observations and equal variance assumptions of a traditional logistic regression model. Therefore, women were nested within households, and households were nested within clusters. Within the cluster, they may have similar characteristics. Hence, multilevel binary logistic regression analysis must take into account the variability between clusters. Since the models were nested. Intra-class correlation coefficient (ICC) and Proportional Change in Variance (PCV) were computed to measure the variation between clusters. The ICC reveals that the variation of poor TT immunization between clusters is calculated as;. Moreover, the PCV reveals the variation in the prevalence of poor TT immunization among pregnant women explained by factors and calculated as; where; Vnull = variance of the initial model, and VA = area/cluster level variance (34). Model fitness was checked using the deviance test and AIC, and the model with the lowest AIC and lowest deviance was selected as the best-fitted model.

First, bivariable multilevel logistic regression analysis was conducted and those variables with a *p* < 0.2 were considered for multivariable analysis. Finally, multilevel binary logistic regression analysis was done to assess the association between TT immunization and individual and community-level factors. Four models were constructed for multivariable multilevel analysis; the null model (without independent variables), mode I (containing only individual-level factors), mode II (containing only community-level factors), and model III (containing both individual and community-level factors) were fitted. In the multivariable model, variables with an adjusted odds ratio (aOR) with a 95% confidence interval (CI) and a *p*-value of <0.05 were considered significantly associated factors of poor TT immunization.

## Results

### Individual and community level factors

A total weighted sample of 7,397 pregnant women were included in this analysis. The median age of the respondents was 28 years (IQR: 24–34) and 87.3% of the women were from rural residence. Of the study participants, 63.1% of the pregnant women had no formal education. More than half (53.9%) of the study participants were not employed and most of (65.6%) the study participants had no media exposure. The majority (90.8%) of the pregnant women were from large central regions. More than two fifths (43.7%) of the pregnant women were from households with poor wealth quantiles (Table 1).

**Table 1:** Individual and community level factors associated with poor TT immunization among pregnant women in Ethiopia.

Variables	Frequency	Percentage (%)
Age in year		
15–24	1,766	23.9
25–34	3,718	50.3
35–49	1,913	25.8
Educational status		
No formal education	4,668	63.1
Primary education	2,095	28.3
Secondary and above	634	8.6
Occupation		
Employed	3,407	46.1
Not employed	3,990	53.9
Household wealth		
Poor	3,230	43.7
Middle	1,552	21
Rich	2,616	35.3
Media exposure		
No	4,849	65.6
Yes	2,548	34.4
Family planning use		
No	4,790	64.8
Yes	2,607	35.2
Number of ANC visits		
No	2,770	37.5
1–3	2,289	30.9
≥4	2,338	31.6
Parity		
Primipara	1,412	19.1
Multipara	3,099	41.9
Grand multipara	2,886	39
Wanted last child		
Wanted then	5,455	73.7
Wanted later	1,277	17.3
Wanted no more	665	9.0
Person who decided on respondents health care		
Self	947	12.8
Joint	4,630	62.6
Husband	1,820	24.6
Resident		
Rural	6,456	87.3
Urban	941	12.7
Community level poverty		
Low	4,484	60.6
High	2,913	39.4
Community-level media exposure		
Low	3,491	47.2
High	3,906	52.8
Community level education		
Low	3,748	50.7
High	3,649	49.3
Region		
Small peripheral	436	5.9
Large central	6,719	90.8
Metropolitan	242	3.3
Distance to the health facilities		
Big problem	4,289	58
Not a big problem	3,108	42

### Regional prevalence of poor TT immunization among pregnant women

**Figure 1:**
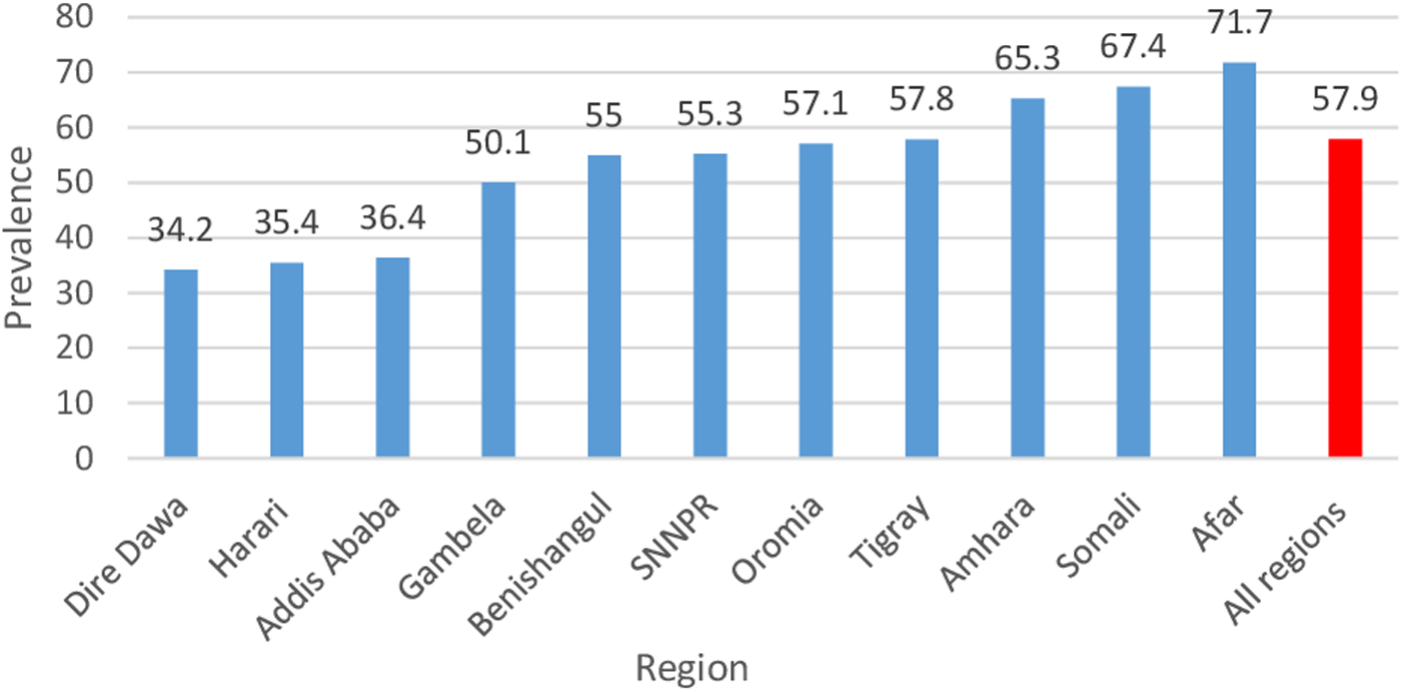
Regional prevalence of TT immunization among pregnant women in Ethiopia, 2016.

The prevalence of poor TT immunization during pregnancy varies across the country. The highest and lowest prevalence of poor TT immunization during pregnancy were observed in the Afar (71.7%) and Dire Dawa (34.2%) regions, respectively (Figure 1).

### Spatial analysis of poor TT immunization

#### Spatial autocorrelation and spatial analysis of TT immunization

**Figure 2:**
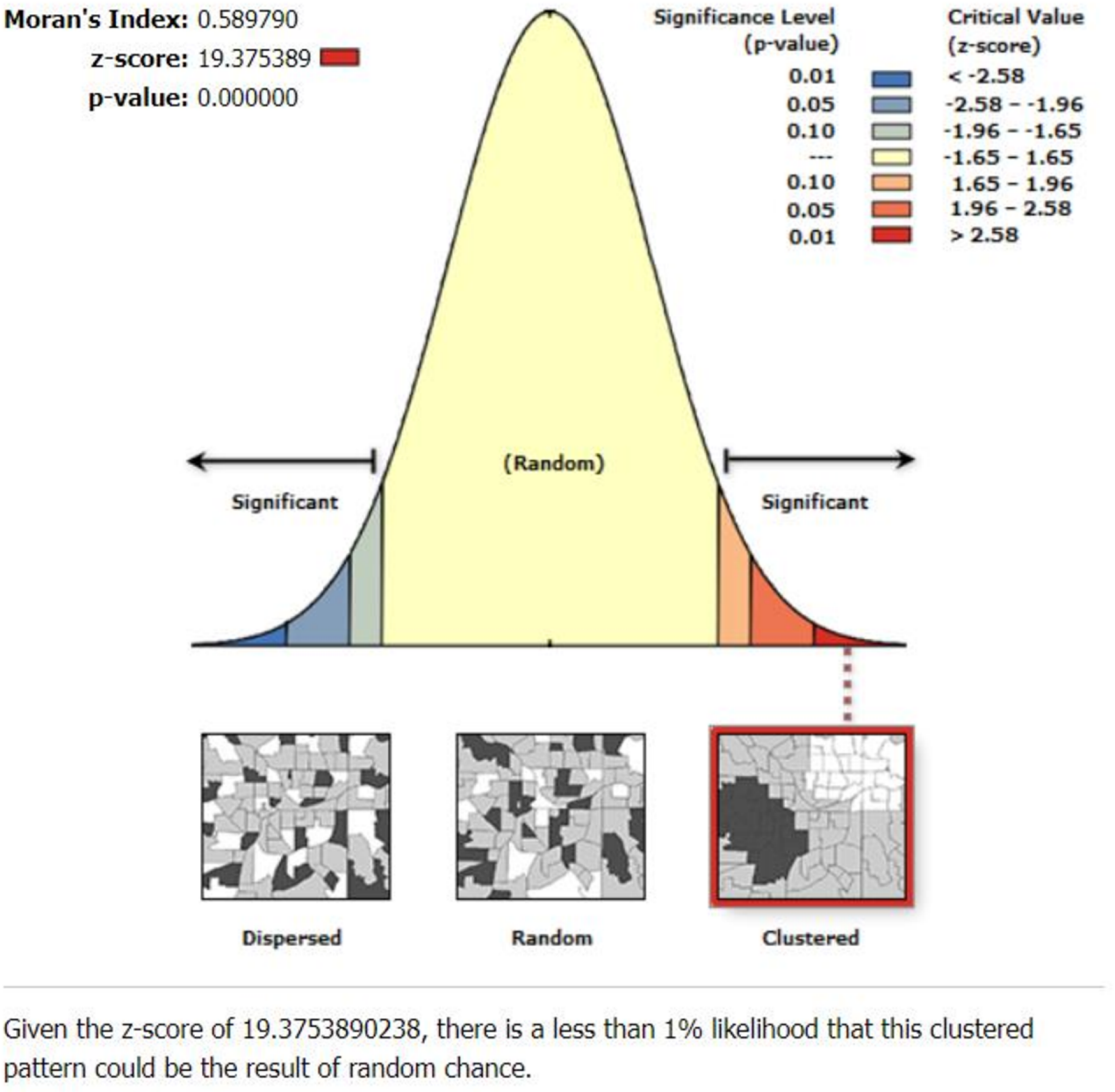
Spatial autocorrelation analysis of poor TT immunization among pregnant women in Ethiopia.

**Figure 3:**
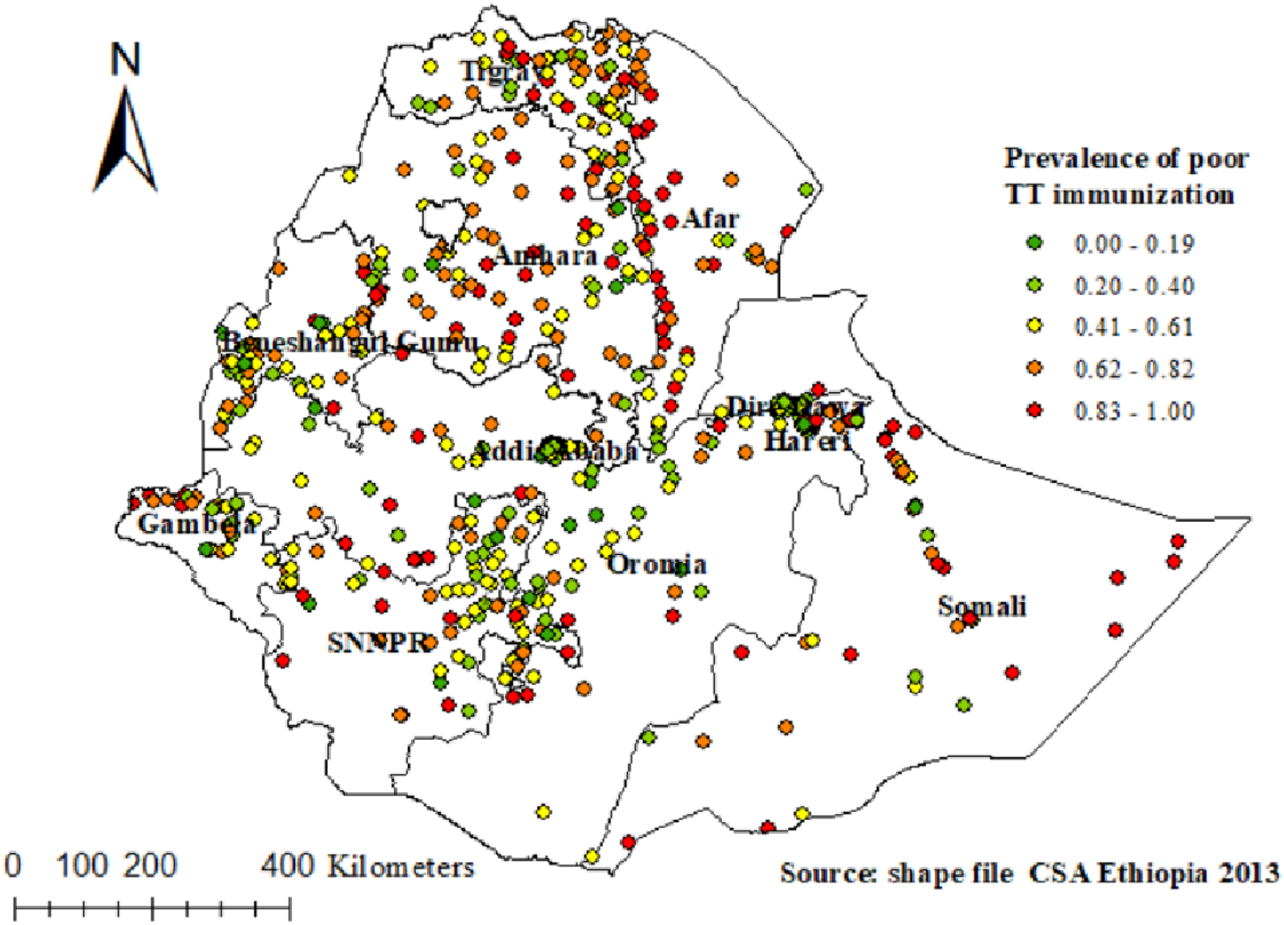
Spatial distribution of poor TT immunization among pregnant women in Ethiopia.

The spatial autocorrelation analysis revealed that the distribution of poor TT immunization was non-random in Ethiopia, with a Global Moran's Index value of 0.59 (*p* < 0.0001) (Figure 2). A higher proportion of poor TT immunization occurred in the east and south of Tigray, the central part of Amhara, West Afar, East Somali, and West Gambella, while low proportions of poor TT immunization were identified in the Addis Ababa, Harari, Dire Dawa, and central Oromia regions (Figure 3).

#### Getis OrdGi statistical analysis of poor TT immunization

**Figure 4:**
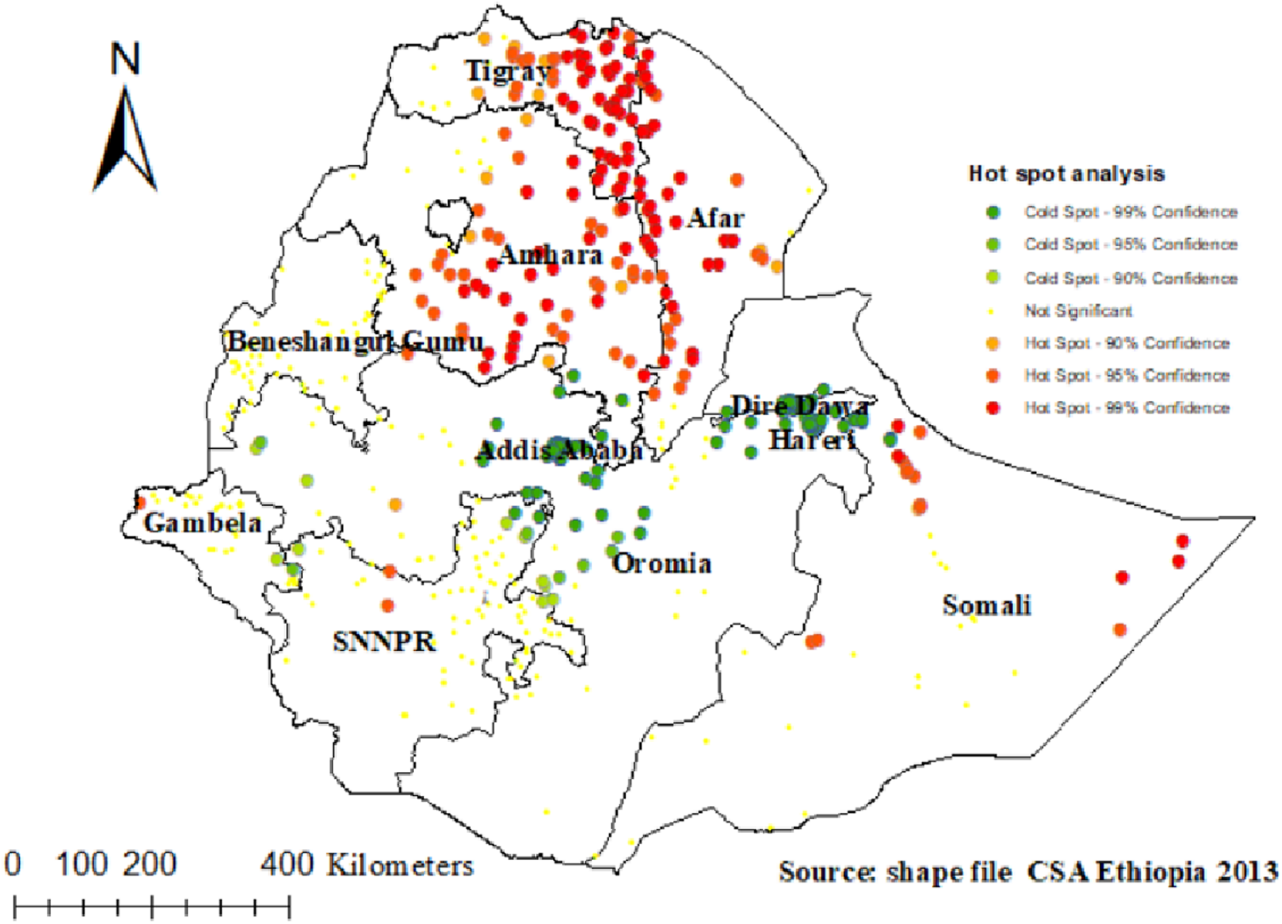
Hot spot analysis of poor TT immunization in Ethiopia.

According to the Getis OrdGi statistics analysis, the majority of Tigray and Amhara regions had significant hotspots (areas with poor TT immunization). In addition, it is located in the west of Afar and northeast of the Somali region. Cold spots (areas with high TT immunization) were found in Addis Ababa, Dire Dawa, Hareri, the northern and central parts of Oromia, and the north-eastern part of SNNPR (Figure 4).

### Kriging interpolation

**Figure 5:**
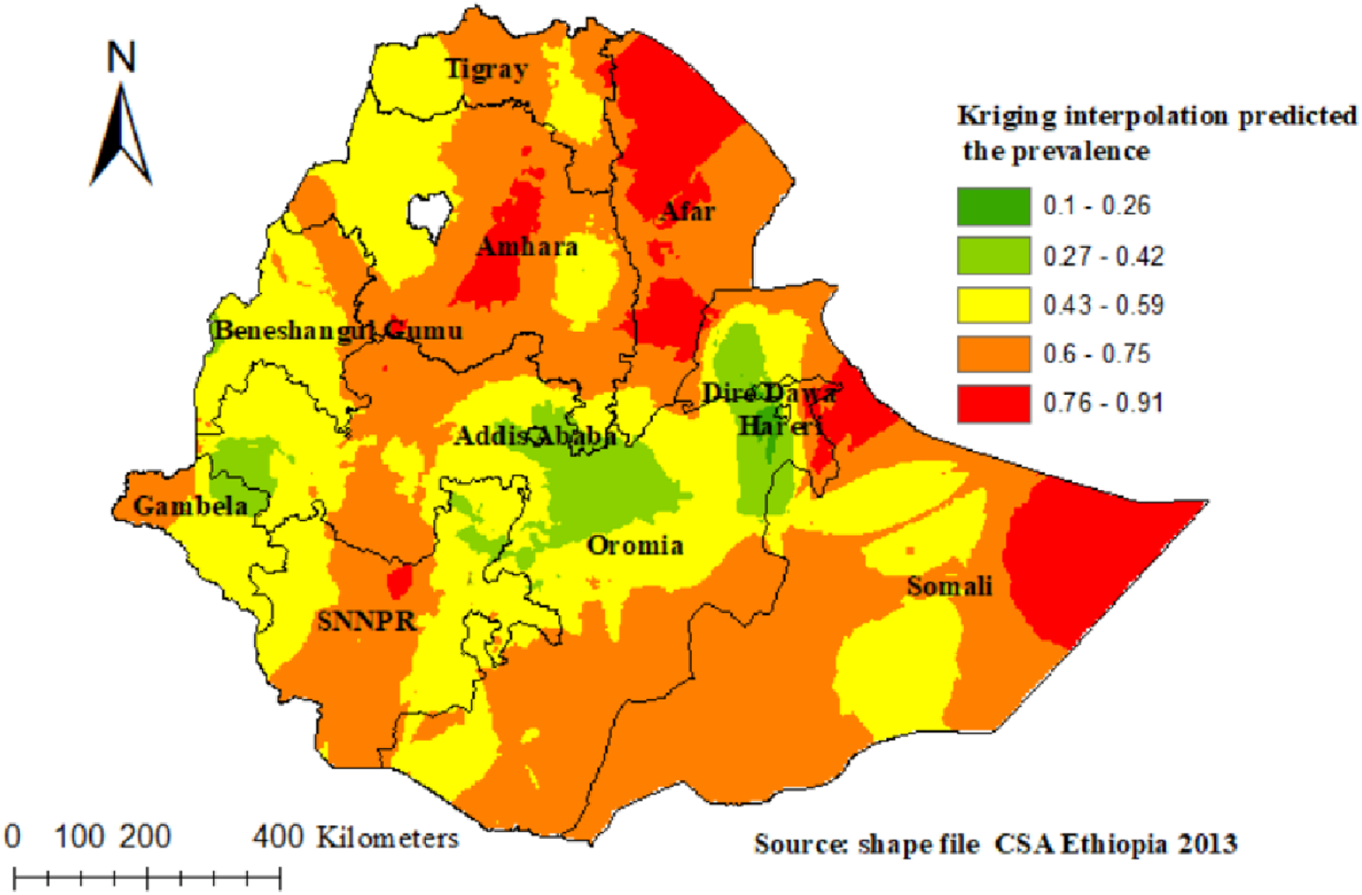
Kriging interpolation of poor TT immunization among pregnant women in Ethiopia.

In the Kriging interpolation; the predicted poor TT immunization coverage were identified in the Eastern and North-eastern part of Somali, northern part of Afar, central and south western part of Amhara, and Northern part of SNNPR regions whereas, the predicted high coverage of TT immunization were identified in the Northeastern part of Gambella, some part of eastern, western and central Oromia, Addis Ababa, Dire Dawa, and northwest Somali regions (Figure 5).

### Spatial Sa Tscan analysis of poor TT immunization

**Figure 6:**
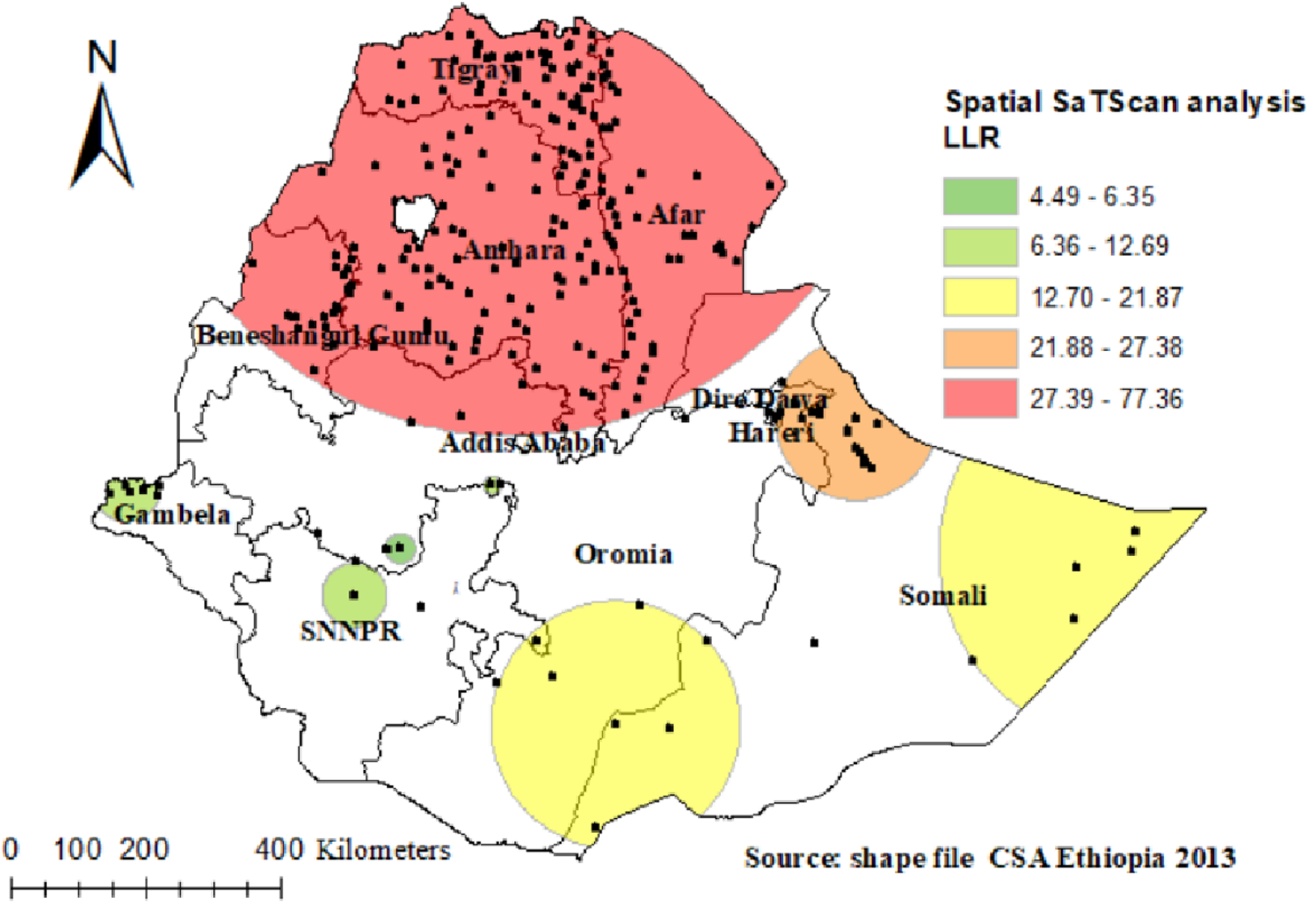
SaTScan analysis of poor TT immunization among pregnant women in Ethiopia.

There were 254 significant clusters identified in the spatial Sa Tscan statistics, of which 209 were primary clusters (most likely). As a result of the survey, the primary clusters were found in the Tigray, Amhara, North Eastern Somali region, Benishangul region, and Oromia region. They were located at 14.122895 N, 38.621010 E of geographic location, with a radius of 561.28 km, with a Relative Risk (RR) of 1.3 and Log-Likelihood ratio (LLR) of 77.36, with *p* = 0.001. According to the study, pregnant women within the spatial window were 1.3 times more likely to have poor TT immunization than pregnant women outside it (Figure 6) (Table 2).

**Table 2:** Sat scan analysis of poor TT immunization among pregnant women in Ethiopia.

Cluster	Enumeration area (cluster) identified	Coordinate/radius	Population	Case	RR	LLR	*P*-value
1 (209)	400, 597, 590, 81, 636, 584, 84, 156, 181, 45, 481, 255, 551, 98, 528, 461, 479, 340, 355, 89, 579, 604, 78, 188, 575, 583, 598, 226, 404, 425, 129, 430, 268, 341, 413, 538, 258, 424, 94, 237, 550, 623, 220, 117, 192, 196, 605, 80, 99, 103, 298, 384, 160, 263, 127, 362, 322, 134, 628, 421, 542, 235, 612, 392, 253, 585, 143, 511, 79, 296, 152, 312, 136, 449, 130, 172, 638, 128, 300, 504, 327, 199, 66, 640, 442, 97, 351, 488, 249, 163, 455, 200, 401, 279, 599, 478, 344, 544, 332, 591, 512, 132, 292, 627, 241, 158, 389, 189, 496, 348, 169, 545, 456, 571, 73, 410, 38, 191, 431, 427, 52, 354, 611, 167, 516, 616, 345, 382, 403, 254, 24, 18, 120, 368, 176, 429, 617, 259, 361, 205, 460, 178, 499, 602, 570, 206, 55, 334, 415, 10, 541, 109, 547, 386, 3, 548, 267, 482, 515, 375, 440, 632, 615, 276, 474, 498, 229, 596, 366, 620, 531, 4, 350, 510, 310, 533, 75, 283, 246, 637, 218, 559, 572, 102, 36, 494, 37, 295, 150, 135, 423, 256, 183, 137, 184, 336, 364, 35, 201, 244, 484, 39, 624, 517, 564, 320, 121, 230, 399	(14.122895 N, 38.621010 E)/561.28 km	2,399	1,605	1.3	77.36	<0.001
2 (23)	527, 277, 568, 33, 22, 116, 439, 64, 57, 239, 210, 573, 8, 251, 214, 186, 566, 1, 622, 436, 212, 501, 307	(9.264633 N, 43.272679 E)/119.44 km	293	226	1.38	27.38	<0.001
3 (5)	269, 630, 629, 77, 146	(7.453674 N, 46.955230 E)/285.37 km	58	55	1.68	21.87	<0.001
4 (8)	377, 394, 422, 7, 34, 289, 480, 398	(5.203234 N, 40.019732 E)/184.64 km	130	106	1.45	18.09	<0.001
5 (6)	309, 536, 370, 618, 233, 69	(8.342595 N, 33.451041 E)/50.46 km	64	55	1.52	12.69	<0.001
6 (2)	338, 76	(6.934084 N, 36.520510 E)/48.00 km	27	26	1.7	11.25	0.012
7 (1)	278	(6.273056 N, 42.688145 E)/0 km	17	171	1.76	9.59	0.032

RR, relative risk; LLR, Log-likelihood Ratio.

### Associated factors of TT immunization

#### Random effect results

Based on the null model, the ICC value for poor TT immunization was 19.6%, which means 19.6% of the variability was explained by group variation, while 80.4% was explained by individual variation. Besides, the higher PCV value (31.63%) in the final model indicates that about 31.63% of the variation of poor TT immunization among pregnant women was attributable to both individual level and community-level factors (Table 3).

**Table 3:** Community-level variability and model fitness for assessment of poor TT immunization among pregnant women in Ethiopia.

Measure of variations	Null model	Model I	Model II	Model III
ICC	19.6	15.2	14.3	13.4
Variance	80.2	63.5	54.7	50.8
PCV (%)	Ref	22.25	27.01	31.63
Model fitness				
Deviance	9,327	8,022.2	9,177.8	8,006.6
AIC	9,331.2	9,102.7	9,013.5	8,207.3

ICC, Intra class correlation coefficient; PCV, proportional change in variance; AIC, Akaike information criterion.

#### Fixed effect result

Number of ANC visits, media exposure, modern family planning use, wealth index, and community-level education were significantly associated with poor TT immunization in the final model (Model III).

Accordingly, pregnant women who had no and 1–3 ANC visits had 10.46 (aOR: 10.46, 95% CI: 8.82, 12.41) and 1.51 (aOR: 1.51, 95% CI: 1.31, 1.73) times higher odds of poor TT immunization than those who had recommended ANC visits, respectively. Pregnant women who had no media exposure had 1.45 (aOR: 1.45, 95% CI: 1.26, 1.67) times more odds to have poor TT immunization than those who had media exposure. Women who did not use modern family planning had 1.28 (aOR: 1.28, 95% CI: 1.11, 1.57) fold higher odds of poor TT immunization as compared with their counterparts. Compared to study participants in the rich households, pregnant women from households classified as poor and moderate status had 1.22 (aOR: 1.22, 95% CI: 1.03, 1.45) and 1.23 (aOR: 1.23, 95% CI: 1.03, 1.47) fold higher odds of poor TT immunization. Moreover, pregnant women who lived in a community with low education had 1.43 (aOR: 1.43, 95% CI: 1.14, 1.80) times higher odds of poor TT immunization as compared with those who lived in a community with high education (Table 4).

**Table 4:** Multi-level mixed-effect logistic regression analysis of individual and community level factors associated with poor TT immunization among pregnant women in Ethiopia.

Variables	Poor TT immunization		COR (95% CI)	Model I AOR (95% CI)	Model II AOR (95% CI)	Model III AOR (95% CI)
Education of the mother	Yes	No				
Secondary education and above	243	391	Ref	Ref		Ref
Primary education	1,039	1,056	1.47 (1.18, 1.83)	1.36 (1.25, 1.48)		0.89 (0.71, 1.13)
No formal education	2,998	1,670	2.31 (1.86, 2.84)	0.99 (0.78, 1.28)		0.97 (0.75, 1.26)
Occupation of the mother						
Employed	1,922	1,485	Ref	Ref		Ref
Not Employed	2,359	1,631	1.12 (0.98, 1.23)	0.98 (0.81, 1.11)		0.95 (0.85, 1.08)
Wealth index						
Rich	1,236	1,379	Ref	Ref		Ref
Middle	936	615	1.57 (1.35, 1.84)	1.06 (0.96, 1.14)		1.23 (1.03, 1.47)*
Poor	2,108	1,222	1.92 (1.67, 2.20)	1.12 (1.03, 1.21)		1.22 (1.03, 1.45)*
Media exposure						
Yes	1,146	1,401	Ref	Ref		Ref
No	3,134	1,715	1.99 (1.75, 2.26)	1.28 (1.19, 1.37)		1.45 (1.26, 1.67)*
Family planning use						
Yes	1,317	1,290	Ref	Ref		Ref
No	2,963	1,827	1.49 (1.33, 1.68)	1,51 (1.38, 1.63)		1.28 (1.11, 1.57)*
Number of ANC visits						
≥4	854	1,485	Ref	Ref		Ref
1–3	1,060	1,229	1.64 (1.43, 1.87)	2.67 (2.35, 2.91)		1.51 (1.31, 1.73)*
None	2,367	403	12.07 (10.27, 14.17)	3.34 (2.98, 3.56)		10.46 (8.82, 12.41)*
Wanted last-child						
Wanted then	3,131	2,324		Ref		Ref
Wanted later	704	573		1.16 (1.05, 1.43)		0.88 (0.76, 1.04)
Wanted no more	445	229		1.23 (1.11, 1.48)		1.19 (0.96, 1.48)
Parity						
Grand multipara	1,806	1,079		Ref		Ref
Multipara	1,751	1,349		1.11 (0.96, 1.27)		1.10 (0.96, 1.26)
Primipara	723	688		1.32 (1.09, 1.58)		1.30 (0.98, 1.56)
Person who decided on respondent health care						
Self	505	443		Ref		Ref
Jointly	2,634	1,996	1.07 (0.91, 1.26)	1.15 (0.96, 1.37)		1.13 (0.95, 1.36)
Husband	1,142	677	1.41 (1.17, 1.69)	1.18 (0.96, 1.44)		1.17 (0.96, 1.43)
Region						
Metropolitan	87	155	Ref		Ref	Ref
Large central	3,910	2,809	2.78 (1.93, 4.01)		1.58 (1.06, 1.36)	1.23 (0.82, 1.84)
Small peripheral	264	152	3.9 (2.53, 6.01)		1.64 (1.04, 1.61)	1.04 (0.64, 1.67)
Residence						
Urban	389	551	Ref		Ref	Ref
Rural	3,890	2,565	2.64 (2.05, 3.40)		1.11 (0.81, 1.51)	0.79 (0.59, 1.05)
Distance to the health facilities						
Not a big problem	1,652	1,457	Ref		Ref	Ref
Big problem	2,628	1,660	1.31 (1.16, 1.48)		1.16 (1.03, 1.31)	0.96 (0.83, 1.11)
Community media exposure						
High	1,729	1,920	Ref		Ref	Ref
Low	2,551	1,196	2.52 (2.05, 3.09)		1.68 (1.34, 12.11)	1.21 (0.96, 1.52)
Community level poverty						
High	1,865	1,048			Ref	Ref
Low	2,416	2,068	0.47 (0.38, 0.59)		0.93 (0.73, 1.18)	1.11 (0.82, 1.36)
Community level education						
High	1,729	1,920	Ref		Ref	Ref
Low	2,551	1,196	2.72 (2.24, 3.32)		1.96 (1.57, 2.45)	1.43 (1.14, 1.80)*

* Statistically significant at *p*-value <0.05, AOR; Adjusted Odds Ratio, COR; Crude Odds Ratio.

## Discussion

According to the present study, the spatial distribution of poor TT immunization among pregnant women in Ethiopia was clustered. In most parts of Tigray and Amhara regions, there were significant hotspots of poor TT immunization. It is also found in western Afar and north-eastern Somali region. Whereas the significant “cold spot” areas with high rates of TT immunization were located in Addis Ababa, Dire Dawa, Harari, central and North-eastern parts of Oromia, and north-eastern part of the SNNPR regions. The possible justification for this difference could be due to the different socio-economic and obstetric-related factors of the study participants. For instance, the majority of the respondents in Addis Ababa, Dire Dawa, and Harari were educated, rich, and had the recommended ANC visits. In addition, most participants who lived in these regions were exposed to the media. Which in turn enhances the pregnant woman's understanding of TT immunization (31, 35).

Poor TT immunization coverage was higher among mothers who did not attend ANC visits compared to those who attended the recommended ANC visits. This finding was supported by studies conducted in Ethiopia (15), Kenya (36), and Pakistan (13). The TT immunization was a routine intervention during the ANC visit, and maternal awareness of continuous immunization may be increased through counselling about TT immunization (15).

Similarly, poor TT immunization was more likely to occur among pregnant women from lower-income households. This is in line with studies conducted in Ethiopia (16) and Bangladesh (37). The possible explanation might be due to economic status, which is one of the significant factors that affect maternal healthcare-seeking behavior, including TT immunization services (16, 38, 39).

Similarly, in this study, community-level education was also significantly associated with poor TT immunization. When compared with their counterparts, people who live in a community with low levels of community education are more likely to have poor TT immunization. Scholars showed that maternal health service utilization is low in a community with low community-level education (16, 40).

Poor TT immunization among those who had no media exposure was higher than for those who had media exposure. This is in line with a study conducted in Ethiopia (16). These findings may be explained by the fact that those exposed to the media have a better understanding of reproductive health services, including TT immunizations, and their advantages (41).

Pregnant women who did not use modern family planning methods were more likely to have poor TT immunization as compared with those who used modern family planning. This finding was supported by studies in Ethiopia (42) and Bangladesh (43). As a result of service integration, health information might be provided during family planning sessions (44).

### Strength and limitation of the study

The current study's key strength was the use of weighted data that were nationally representative.

As a result, the results of the current study can be applied nationally. In addition, we also identify similar and statistically significant areas with a high cluster of TT immunization by using both ArcGIS and Sat Scan statistical tests. Due to the cross-sectional nature of the study, it does not show the cause-and-effect relationship between the outcome and independent variables. Because the current study relied on secondary data, some essential variables, such as respondents' knowledge of TT vaccines and sociocultural factors, were missing.

## Conclusions

Poor tetanus toxoid immunization among pregnant women varies in Ethiopia. It was highest in the East and South Tigray, the central part of Amhara, West Afar, East Somali, and West Gambella. Number of ANC visits, media exposure, wealth index, family planning use, and community-level education were significantly associated with poor TT immunization. Therefore, TT immunization should be improved in the identified hotspot areas by designing locally targeted public health interventions. Public health interventions like promoting the dissemination of information related to TT immunization in the media, women's education, and modern family planning use have the potential to improve women's awareness of TT vaccines, which in turn will improve TT immunization.

## Data Availability

The raw data supporting the conclusions of this article will be made available by the authors, without undue reservation.
